# Potency, Safety, and Pharmacokinetic Profiles of Potential Inhibitors Targeting SARS-CoV-2 Main Protease

**DOI:** 10.3389/fphar.2020.630500

**Published:** 2021-02-01

**Authors:** Hylemariam Mihiretie Mengist, Daniel Mekonnen, Ahmed Mohammed, Ronghua Shi, Tengchuan Jin

**Affiliations:** ^1^ Department of Obstetrics and Gynecology, The First Affiliated Hospital of USTC, Division of Life Sciences and Medicine, University of Science and Technology of China, Hefei, China; ^2^ Hefei National Laboratory for Physical Sciences at Microscale, Division of Life Sciences and Medicine, The CAS Key Laboratory of Innate Immunity and Chronic Disease, School of Basic Medical Sciences, University of Science and Technology of China, Hefei, China; ^3^ Department of Medical Laboratory Science, College of Health Sciences, Debre Markos University, Debre Markos, Ethiopia; ^4^ Department of Medical Laboratory Science, College of Health Science and Medicine, Bahir Dar University, Bahir Dar, Ethiopia; ^5^ CAS Center for Excellence in Molecular Cell Science, Chinese Academy of Science, Shanghai, China

**Keywords:** potency, safety, pharmacokinetics, inhibitors, SARS-CoV-2, main protease, COVID-19

## Abstract

Effective, safe, and pharmacokinetically suitable drugs are urgently needed to curb the ongoing COVID-19 pandemic. The main protease or 3C-like protease (M^pro^ or 3CL^pro^) of SARS-CoV-2 is considered an important target to formulate potent drugs corresponding to its crucial role in virus replication and maturation in addition to its relatively conserved active site. Promising baseline data on the potency and safety of drugs targeting SARS-CoV-2 M^pro^ are currently available. However, preclinical and clinical data on the pharmacokinetic profiles of these drugs are very limited. This review discusses the potency, safety, and pharmacokinetic profiles of potential inhibitors of SARS-CoV-2 M^pro^ and forward directions on the development of future studies focusing on COVID-19 therapeutics.

## Introduction

Coronavirus disease 19 (COVID-19), caused by severe acute respiratory syndrome coronavirus 2 (SARS-CoV-2), is causing significant social, economic, and political disturbances worldwide. The number of cases is above seventy-nine million with a toll of death surpassing 1.74 million (https://www.worldometers.info/coronavirus/) as of December 24, 2020. The presence of asymptomatic carriers, various modes of transmission, limitation of point-of-care diagnostic facilities especially in resource-limited countries, and lack of globally approved vaccines and antiviral drugs (Cascella et al., 2020; Covid et al., 2020; Mekonnen et al., 2020; Patel et al., 2020; Wang et al., 2020b; Zhang et al., 2020d) are among others worsening the challenge.

Although remdesivir is currently approved by the FDA of the USA for COVID-19 treatment (Beigel et al., 2020), conflicting clinical results have been reported. Remdesivir helps fast recovery of moderate and severely affected patients but its clinical effect on nonmechanically ventilated severely affected patients is optimal (Elsawah et al., 2020). This indicates that the treatment of COVID-19 is still medically unmet requiring further efforts. Currently, patient management is primarily dependent on symptomatic treatment and respiratory support including intensive care in case of complicated disease (Cascella et al., 2020; Chen et al., 2020; Gattinoni et al., 2020). Fifteen drugs (chloroquine, hydroxychloroquine, lopinavir, ritonavir, nafamostat, camostat, famotidine, umifenovir, nitazoxanide, ivermectin, corticosteroids, tocilizumab, sarilumab, bevacizumab, and fluvoxamine) are under clinical trial (Shaffer, 2020) for COVID-19 treatment. In addition, several antivirals (bemcentinib, chloroquine and hydroxychloroquine, lopinavir boosted with ritonavir and remdesivir) and immune modulators (anakinra and canakinumab, azithromycin, brensocatib, convalescent plasma, corticosteroids, interferon beta, ruxolitinib, mesenchymal stromal cells and sarilumab and tocilizumab) are also being considered for clinical use (Connelly, 2020).

Promising drugs targeting SARS-CoV-2 M^pro^ have been under investigation demonstrating efficient binding and potential antiviral activities. The main protease is a key enzyme important for viral replication and maturation (Ziebuhr et al., 2000; Jain and Mujwar, 2020). Besides, the M^pro^ has enhanced enzymatic activity and there are no human proteases reported yet having similar specificity with it (Hilgenfeld, 2014; Zhang et al., 2020a, Zhang et al., 2020b, Zhang et al., 2020c) strengthening its preference of being a potential drug target. Several compounds including new drugs, known antivirals, and repurposed broad-spectrum drugs showed effective inhibition of SARS-CoV-2 M^pro^ with promising antiviral activities.

**FIGURE 1:**
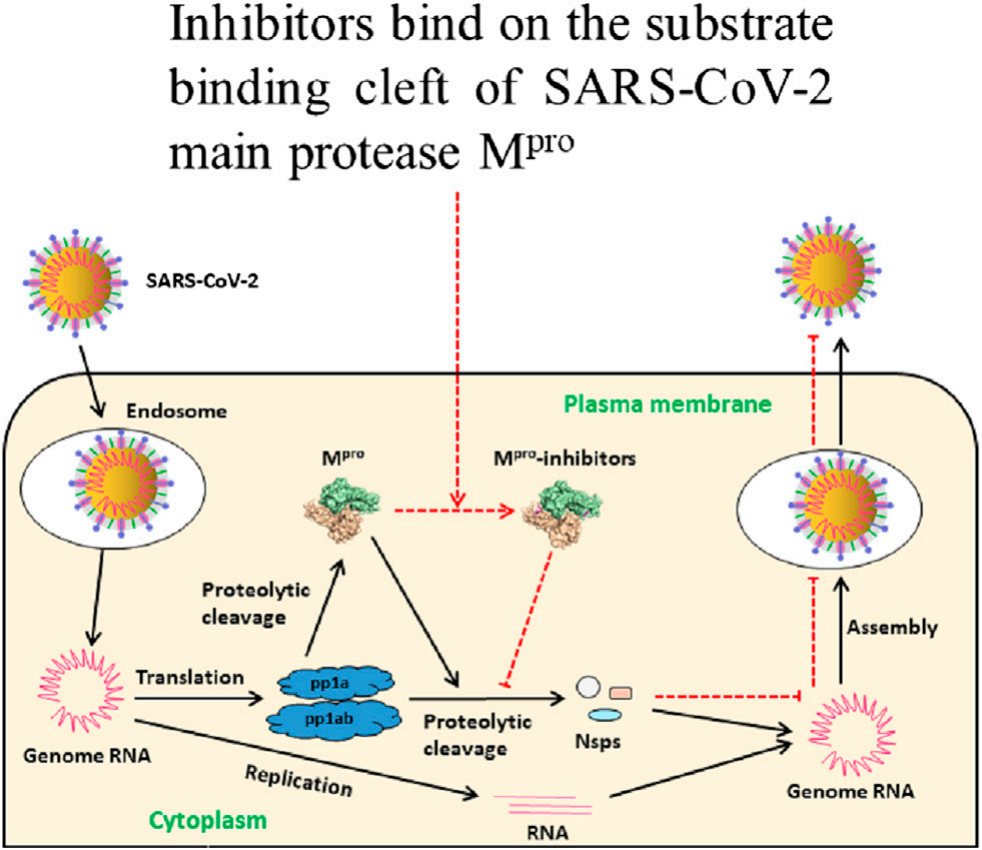
Schematic illustration of inhibitors of M^pro^ preventing SARS-CoV-2 replication. After entering into the host cell, SARS-CoV-2 releases its genomic RNA. Translation produces polyproteins pp1a and pp1ab which are cleaved to M^pro^ and nonstructural proteins (nsps). M^pro^ is involved in the production of nsps and virion maturation. These proteins are essential for assembling the viral replication transcription complex (RTC) to engage in RNA synthesis. M^pro^ inhibitors bind on its substrate-binding cleft resulting in inactivation with subsequent failure of virion assembly. Eventually, host cells fail to release the new intact virions and thus new infection is inhibited, modified from Mengist et al. (2020).

So far, peptidomimetic alpha ketoamide inhibitors (**13a**, **13b**) (Zhang et al., 2020a, Zhang et al., 2020b; Mengist et al., 2020), Michael acceptor N3 (Jin et al., 2020a), carmofur (Jin et al., 2020a; Jin et al., 2020b), ebselen (Jin et al., 2020a; Sies and Parnham, 2020), aldehyde-based compounds **11a** and **11b** (Dai et al., 2020), and **6e** (Rathnayake and Zheng, 2020), clinically approved anti-Human immunodeficiency virus (HIV) drugs lopinavir/ritonavir (Liu and Wang, 2020), antiplatelet drug dipyridamole (Li et al., 2020c; Liu et al., 2020), anti-Hepatitis C virus (HCV) drug boceprevir, GC-376, calpain inhibitors (II, XII), and GC-373 (Choy et al., 2020; Ma et al., 2020; Vuong and Khan, 2020) are among the most promising drugs reported exhibiting effective *in vitro* and *in vivo* antiviral activity. These drugs bind on the substrate-binding cleft of the M^pro^ and inhibit its activity with the subsequent halting of virus replication and infection (Figure 1). However, the clinical outcome of these drugs in humans is not determined yet. Further, the antiviral activity and safety of several drugs are heterogeneous and the results of various studies are not collated together yet. Summarizing the potency, safety, and pharmacokinetic profiles of these drugs could be crucial to recommend the best ones for further investigation. Therefore, this review aims to evaluate potential inhibitors of SARS-CoV-2 M^pro^ concerning their antiviral activity (potency), safety, and pharmacokinetic profiles summarized in Table 1.

**TABLE 1:** Description of the potency, safety, and pharmacokinetic profiles of drugs targeting SARS-CoV-2 M^pro^.

Drug	Potency (EC_50,_ µM)	Safety (CC_50,_ µM)	Pharmacokinetic profile			Remark
			T_1/2_ (hours)	C_max_ (ng/ml)	Clearance (ml/min/kg)	
13a (Zhang et al., 2020a, Zhang et al., 2020b.)	4–5^c^		1.0 ± 0.1	334.5 ± 109.2	565.6 ± 61.0	Administered in CD-1 mice (20 mg/kg)^sc^
13b (Zhang et al., 2020a, Zhang et al., 2020b.)	4–5^c^		1.8 ± 0.5	126.2 ± 31.0	131.6 ± 26.0	Administered in CD-1 mice (3 mg/kg)^sc^
Ebselen (Jin et al., 2020a; Masumoto et al., 1997.)	4.67^c^	>100^c^	2.1	14780^or^		Activity in rats
N3 (Jin et al., 2020a.)	16.77	>130				Activity in Vero cells
Cinanserin (Jin et al., 2020a.)	20.61	>200				Activity in Vero cells
Carmofur (Jin et al., 2020b.)	24.0 ± 3.61	133 ± 12				Activity in Vero E6 cells
11a (Dai et al., 2020.)	0.53 ± 0.01^c^	>189	4.27 ± 1.23^ip^	2,394 ± 288^ip^	17.4 ± 2.76^iv^	Administered in CD-1 mice (5 mg/kg)
11b (Dai et al., 2020.)	0.72 ± 0.09^c^	>139	5.21 ± 1.35^ip^	3,019 ± 665^sc^	20.6 ± 2.0^iv^	Administered in CD-1 mice (5 mg/kg)
6e (Rathnayake and Zheng, 2020.)	0.15	63.3 ± 2.3				Activity in Vero E6 cells
GC-373 (Vuong and Khan, 2020.)	1.50 ± 0.30	>200				Activity in Vero E6 cells
GC-376 (Vuong and Khan, 2020.)	0.90 ± 0.20	>200				Activity in Vero E6 cells
Boceprevir (Ma et al., 2020; Treitel et al., 2012.)	1.95 ± 1.62^c^	>100^c^	6.51	914	157^#^	Activity in severe hepatic failure patients
Telaprevir (Gammeltoft et al., 2020; Garg et al., 2013.)	40^c^	>432^c^	3.8 ± 0.8	1899		Activity in healthy human volunteers
Narlaprevir (Arasappan et al., 2010; de Bruijne et al., 2010; Isakov et al., 2016.)	0.04	269^c^	9.3	1,630		Activity in chronic HCV patients/cirrhosis
Grazoprevir (Gammeltoft et al., 2020.)	20	133				Activity in Huh7.5 cells
Simeprevir (Gammeltoft et al., 2020; Ouwerkerk-Mahadevan et al., 2015.)	14^c^	33^c^		2,588		Activity in renally impaired patients
CI-II (Ma et al., 2020.)	3.70 ± 0.69	>100				Activity in Vero 76 cells
CI-XII (Ma et al., 2020.)	0.78 ± 0.37	>100				Activity in Vero 76 cells
GC-376 (Ma et al., 2020.)	3.13 ± 1.01	>100				Activity in Vero 76 cells
GC-376 (Hung et al., 2020)	0.91 ± 0.03	>100				Activity in Vero E6 cells
UAWJ246 (Sacco et al., 2020.)	4.61 ± 2.63	>250				Activity in Vero cells
UAWJ247 (Sacco et al., 2020.)	2.06 ± 0.93	184 ± 4.8				Activity in Vero cells
UAWJ248 (Sacco et al., 2020.)	11.10 ± 4.20	>250				Activity in Vero cells
Ebselen (Brown et al., 2020.)	0.026 ± 0.009					FRET biosensor result without Triton X-100
4-CMBA (Brown et al., 2020.)	0.095 ± 0.007					FRET biosensor result without Triton X-100
Ementine (Choy et al., 2020b.)	0.46					Activity in Vero E6 cells
Homorringtonine (Choy et al., 2020b.)	2.55					Activity in Vero E6 cells
Baicalin (Su et al., 2020.)	10.27	>200				Activity in Vero E6 cells
Baicalein (Su et al., 2020.)	1.69	>200				Activity in Vero E6 cells
Remdesivir (Hu et al., 2020; Humeniuk et al., 2020; Söriigel et al., 2020; Wang et al., 2020a.)	0.77^c^	>100^c^	1.05^if^	4420^if^	719^if^	Activity in healthy humans
			0.80 ± 0.08	905^£^	1740 ± 162	Administered in CD-1 mice (20 mg/kg)^iv^
			1.1^iv^	19800^iv^	257^iv^	Administered in a COVID-19 patient (225 mg/kg)
Nafamostat (Wang et al., 2020a.)	22.50^c^	>100				Activity in Vero E6 cells
Lopinavir (Dandache et al., 2007; Gorbalenya et al., 2020; Zhang et al., 2020c.)	0.019 ± 0.001^c^	80.82^c^	13.81	2000	3.81^#^	Activity in a pharmacokinetic model of white and Chinese populations
Ritonavir (Murphy et al., 2001; Zhang et al., 2020c.)	0.07*	94.7^c^	3.40 ± 0.96	710		Activity in HIV-1 patients
Ritonavir (Gorbalenya et al., 2020)			2.7 ± 1.09	1,370	0.25 ± 0.09^#^	Activity in healthy volunteers
Rupintrivir (Zhang et al., 2020c)		>100^c^				Activity in Vero E6 cells
AG7404 (Zhang et al., 2020c)		>400^c^				Activity in Vero E6 cells
Imatinib (Dyall et al., 2014; Weston et al., 2020.)	9.82	>30.86				Activity in Vero E6 cells
Ribavirin (Cinatl et al., 2003.)	>1000^$^	>1000^$^				Activity in Vero cells

**Key**: 4-CMBA: 4-chloromercuribenzoic acid, FRET: fluorescence resonance energy transfer, ip: intraperitoneally, iv: intravenously, sc: subcutaneously, if: infusion, or: orally c: experiment done in cell culture, *: measured in µg/mL, #: measured in L/h/kg, $: measured in mg/L, and £: measured in nmol/kg.

The chemical formula, IUPAC name, and the chemical structure of potential SARS-CoV-2 M^pro^ inhibitors are described in Supplementary Table S1.

### Potency

Many potential drugs have been showing effective antiviral activity *in vitro* and *in vivo* (Ullrich and Nitsche, 2020). Pyridones containing peptidomimetic alpha-ketoamide inhibitors **13a** and **13b** are amongst the promising drugs that exhibited strong SARS-CoV-2 inhibition. In human Calu-3 lung cells infected with SARS-CoV-2, **13b** showed good inhibitory activity with a half-maximal effective concentration (EC_50_) value of 4–5 µM (Zhang et al., 2020a, Zhang et al., 2020b). However, the *in vivo* potency and safety of **13a** and **13b** are not reported yet, which needs further investigation. Ebselen and N3 are other promising drugs targeting M^pro^. In Vero cells, ebselen and N3 displayed effective inhibition of SARS-CoV-2 replication and infection. Ebselen demonstrated more potency (EC_50_ < 5 µM) over N3 as the antiviral activity of N3 was moderate with a relatively higher EC_50_ value >16 µM (Jin et al., 2020a).

Aldehyde-based drugs **11a** and **11b** were synthesized being suitable to bind and inhibit SARS-CoV-2 M^pro^ with respective 100% and 96% *in vitro* inhibition of the M^pro^ at 1 µM. Regarding their antiviral activity, plaque assay in cell culture showed that **11a** and **11b** demonstrated excellent anti-SARS-COV-2 infection activity with very low EC_50_ values <1 µM (Dai et al., 2020). Carmofur is an antineoplastic drug currently considered for COVID-19 treatment. Carmofur is reported to moderately inhibit SARS-CoV-2 infection in Vero E6 cells with an EC_50_ value of >20 µM (Jin et al., 2020b).

Rathnayake et al. (Rathnayake and Zheng, 2020) demonstrated the potency of compounds in SARS-CoV-2 infected Vero E6 cells via targeting the main protease. Accordingly, the synthesized compounds showed effective inhibition of virus replication with EC_50_ values between 0.15 and 0.9 µM where compound **6e** exhibited the most potent activity. The activity of synthesized compounds was also confirmed by the significant difference in the virus plaque-forming units (PFU) observed in the presence and absence of M^pro^ inhibitors in cell culture. Plaque assays and virus reduction assays indicated that GC-373 and GC-376 demonstrated effective inhibition and reduction of SARS-CoV-2 RNA copies in Vero E6 cells with EC_50_ values between 0.9 and 1.5 μM. Comparatively, GC-376 showed stronger inhibitory activity over GC-373 evidenced by a very low EC_50_ value below 1 μM (Vuong and Khan, 2020).

According to Ma et al. (2020), boceprevir, GC-376, and calpain inhibitors II and XII also demonstrated effective inhibition of SARS-CoV-2 replication in Vero 76 cells. The FDA approved HCV drug boceprevir that showed an effective viral reduction with an EC_50_ value below 2 µM while calpain inhibitor II and GC-376 demonstrated a higher EC_50_ value above 3 µM. Among these, calpain inhibitor XII exhibited the most potent antiviral activity against SARS-CoV-2 with a very low EC_50_ value below 1 µM. Further, GC-376 and boceprevir showed effective inhibition of SARS-CoV-2 replication in Vero cells. GC-376 exhibited a strong inhibition potency more than boceprevir (average EC_50_ values: 0.70 µM for GC-376 and 15.57 µM for boceprevir). The authors reported that a combination of 1 µM GC-376 and 1 µM remdesivir can completely inhibit SARS-CoV-2 *in vitro* replication (Choy et al., 2020).

Besides, GC-376 was also reported to effectively inhibit SARS-CoV-2 infection in Vero E6 cells (Hung et al., 2020) where a plaque assay stated a 0.49 ± 0.35 μM EC_50_ value of GC-376. GC-376 analogs (UAWJ246, UAWJ247, and UAWJ248) also produced effective inhibition of SARS-CoV-2 in Vero cells where UAWJ247 demonstrated the strongest inhibition (Sacco et al., 2020). Another study also reported excellent potency of GC-376 against SARS-CoV-2 with an EC_50_ value of 0.91 ± 0.03 μM in Vero E6 cells (Hung et al., 2020). This drug (GC-376) is known to exhibit a strong potency against several other coronaviruses in cell lines (Kim et al., 2012). Brown et al. (2020) used a fluorescence resonance energy transfer (FRET) biosensor to evaluate the potency of 65 compounds against SARS-CoV-2 M^pro^. Among these, ebselen and 4-chloromercuribenzoic acid demonstrated the strongest virus inhibition in the presence and absence of Triton X-100. Baicalin and baicalein are noncovalent nonpeptidomimetic compounds exhibiting effective binding and inhibition of SARS-CoV-2 M^pro^ with baicalein showing the strongest potency (EC_50_ value <2 µM) close to chloroquine and remdesivir (Su et al., 2020). An in silico study predicted remdesivir and nafamostat bind on the catalytic dyad of the M^pro^ (Chakraborti et al., 2020) with potent antiviral activities in cells (Wang et al., 2020a).

### Safety

The *in vivo* safety of proposed drugs for COVID-19D targeting the M^pro^ is not explicitly reported. But the *in vitro* half cytotoxic concentration (CC_50_) values of some drugs are reported. Drugs **11a** and **11b** showed good safety to cells with a CC_50_ value of >100 µM *in vitro*. Specifically, **11a** showed no obvious toxicity in rats and dogs given at different doses for seven days (Dai et al., 2020). Studies reported that ebselen has very low toxicity in rats (Renson et al., 1982) and is safe for humans in clinical trials (Lynch and Kil, 2009; Masaki et al., 2016; Kil et al., 2017). N3 and cinanserin are also reported to be safe to Vero cells with a CC_50_ value of >100 µM with cinanserin exhibiting comparatively low toxicity (CC_50_ value > 200 µM) (Jin et al., 2020a). Carmofur also demonstrated low toxicity in Vero E6 cells with an average CC_50_ value of >133 µM (Jin et al., 2020b). In cell culture, although reported to have high potency, **6e** exhibited relatively higher toxicity to cells with a CC_50_ value below 100 µM. On the contrary, other compounds (**6c**, **6h,** and **6j**) demonstrated an acceptable level of toxicity with CC_50_ values > 100 µM (Rathnayake and Zheng, 2020).

Feline coronavirus drugs targeting SARS-CoV-2 M^pro^ (GC-373 and GC-376) demonstrated very low toxicity in Vero E6 cells with CC_50_ values above 200 µM (Vuong and Khan, 2020). Boceprevir, GC-376, and calpain inhibitors II and XII also demonstrated acceptable level of toxicity with CC_50_ values above 100 µM in cell culture (Ma et al., 2020). A study showed that boceprevir and GC-376 did not cause obvious *in vitro* toxicity to Vero cells (Choy et al., 2020). Interestingly the CC_50_ value of GC-376 is higher in Vero E6 cells indicating its low toxicity (Hung et al., 2020). GC-376 analogs UAWJ246, UAWJ247, and UAWJ248 also demonstrated very low toxicity to Vero cells where UAWJ246 and UAWJ248 displayed a CC_50_ value > 250 µM while the CC_50_ value of UAWJ247 was between 179 and 189 µM (Sacco et al., 2020). The safety of the anticipated drugs should be elaborately investigated for a better understanding of their toxicity properties.

Baicalin and baicalein demonstrated very low cytotoxicity in Vero E6 cells with CC_50_ values > 200 µM (Su et al., 2020). Thimerosal, phenylmercuric acetate, hematoporphyrin, chloranil, plumbagin, Evans blue, and Chicago sky blue showed effective inhibition against SARS-CoV-2 M^pro^ (Coelho et al., 2020). Earlier, the safety of plumbagin, Evans blue, and Chicago sky blue measured by median lethal dose (LD_50_) was reported to be 16, 340, and 2,260 mg/kg administered through different routes in mice/rats (Weinberg et al., 1951; Krishnaswamy and Purushothaman, 1980; Balzarini et al., 1986). Known drugs, remdesivir and nafamostat, also showed acceptable cytotoxicity in cell culture (Wang et al., 2020a; Wang et al., 2020b); however, with increasing clinical application, remdesivir is showing adverse effects in COVID-19 patients (Fan et al., 2020). Lopinavir/ritonavir monotherapy was also found to be toxic with poor clinical effects in mild/moderate COVID-19 patients (Li et al., 2020b).

### Pharmacokinetic Profiles

Studies reporting the *in vivo* pharmacokinetic properties of prospective COVID-19 drugs targeting SARS-CoV-2 M^pro^ are scarce. Alpha-ketoamide drug **13a** demonstrated good metabolic stability with low intrinsic clearance rates in mouse and human microsomes. When administered subcutaneously in CD-1 mice with different doses, **13b** showed higher plasma half-life (T_1/2)_ and a lower clearance rate than **13a**. On the other side, **13a** was better concerning the average amount in plasma with a higher plasma maximal concentration (C_max_) value above 334 ng/ml (Zhang et al., 2020a, Zhang et al., 2020b).

More data are available for drugs **11a** and **11b** which exhibited different pharmacokinetic properties when administered in different routes in CD-1 mice. **11a** showed better plasma T_1/2_ when administered to mice intraperitoneally than intravenously (5 mg/kg). Comparatively, **11a** displayed a high C_max_ and a good bioavailability when administered intraperitoneally. Its metabolic stability, measured by the rate of clearance (ml/min/kg), was also good. **11b** also showed good pharmacokinetic properties when administered intraperitoneally (20 mg/kg), subcutaneously (5 mg/kg), and intravenously (5 mg/kg). More specifically, **11b** showed good bioavailability when given both intraperitoneally and subcutaneously (Dai et al., 2020).

When administered intravenously, **11b** showed faster clearance and shorter half-life indicating the suitability of **11a** through this route. Further pharmacokinetic assessment of **11a** showed, when administered intravenously (10 mg/kg) to SD rat, that it demonstrated low clearance (4.01 ml/min/kg), long T_1/2_ (7.6 h), and high 3-min maximum concentration (81,500 ng/ml). Conversely, **11a**, when administered intravenously (5 mg/kg) to beagle dog, exhibited higher clearance (5.80 ml/min/kg), shorter T_1/2_ (5.5 h), and lower 3-min maximum concentration (21,900 ng/ml) (Dai et al., 2020) indicating better pharmacokinetic profiles in SD rat administered at high dose than beagle dog. Further, the authors also reported that **11a** exhibited no obvious toxicity in rats and dogs administered intravenously at appropriate doses. It is considered, due to safety issues, that intravenous administration is more appropriate where **11a** exhibited interesting pharmacokinetic properties. In a single-ascending-dose randomized controlled study, remdesivir showed different pharmacokinetic profiles. When administered as a 2-h infusion (225 mg), remdesivir showed a low clearance rate, good half-life, and high C_max_ (Humeniuk et al., 2020).

Several clinically approved drugs showed effective binding on SARS-CoV-2 M^pro^ with possible antiviral activities. Among these, HCV NS3/4A protease inhibitors (sovaprevir, vaniprevir, glecaprevir, boceprevir, simeprevir, paritaprevir, danoprevir, and grazoprevir) (Bafna et al., 2020), HIV protease inhibitors [nelfinavir (Xu et al., 2020) and lopinavir/ritonavir (Nukoolkarn et al., 2008)], immune modulators (vinflunine, vindesine, and topotecan) (Chakraborti et al., 2020), and other drugs including colistin (antibiotic), valrubicin (antitumor), icatibant (indicated for hereditary angioedema), bepotastine (prescribe for rhinitis), caspofungin (antifungal), perphenazine (antipsychotic) (Liu and Wang, 2020), bromocriptine (a dopamine antagonist), ergotamine (antimigraine), bictegravir (antiviral), antibacterial agents (oxytetracycline, tigecycline, and ceftolozane) (Chakraborti et al., 2020), viz. D2 receptor antagonist, HMG-CoA inhibitors, HIV reverse transcriptase and protease inhibitors, anticancer agents, folate inhibitors, and imatinib (Balaramnavar et al., 2020) showed effective binding on the M^pro^. Although their suitability in COVID-19 patients is to be determined, the potency, safety, and/or pharmacokinetic profiles of known drugs inhibiting SARS-CoV-2 M^pro^ are reported before, which is briefly described in Table 1.

## Discussion and Perspectives

Determining the potency, safety, and pharmacokinetic profiles of drugs and applying it into clinical practice is the ultimate aim of drug discovery. Determining the binding affinity and efficiency of the anticipated drugs with the target, evaluating its target inhibitory activity, and assessing its role in curbing infection *in vitro* are all early stages of the process of drug discovery. The drug should be evaluated in model organisms *in vivo* and should be tested in a cohort of humans under clinical trials which is the most challenging step to achieve. The physiological process in humans is quite complex which affects the pharmacokinetic and pharmacodynamic properties of the anticipated drugs. The final goal in therapeutic medical research is to find an effective, safe, and pharmacokinetically suitable drug with minimum side effects on human tissues. This is quite challenging and that is why, although a couple of months passed, there is no globally approved specific antiviral drug yet to treat the COVID-19 pandemic.

Scientists have been investigating the potency and safety of old and new drugs through studying the ability of the drugs to specifically bind and inhibit target proteins and control virus replication. The emergence of tremendous publications on COVID-19 therapy is proof of the ongoing efforts in discovering potential drugs (Bein et al., 2020; Li et al., 2020a; McCreary and Pogue, 2020; Sanders et al., 2020). However, only a small fraction of studies presented data on the preclinical and clinical potency, safety, and pharmacokinetic properties of drugs where the progress is more infant in drugs targeting SARS-CoV-2 M^pro^ as most studies lack experimental validation where only scientific simulation data are available.

Urgent COVID-19 therapeutic options are desired by the community (de Almeida et al., 2020). In this regard, based on previous therapeutic experience with SARS-CoV and MERS-CoV, there has been a substantial inquisitiveness in the repurposing of approved antiviral drugs (for example, drugs used to treat HIV, HBV, HCV, filoviruses, and influenza) and development of new drugs for COVID-19 (Artese et al., 2020; Liu and Wang, 2020). Apart from the ongoing struggles in searching for effective drugs for COVID-19, challenges are facing these efforts (Ghaebi et al., 2020). Among these, urgency is of significant factor which is exacerbated by the time-consuming and expensive nature of the data acquisition process in physical experiments. Intriguingly, the application of computational simulations and drug repurposing programs significantly alleviates the problem through providing basic data; however, whether these drugs pass clinical trials is another headache to the scientific world which puts the progress of finding clinically applicable COVID-19 drugs at its early stage. A mutant coronavirus was reported on November 5, 2020 in mink populations in Denmark which can spread to humans (Lesté-Lasserre, 2020). Besides, a recent study by Hou et al. (Wu et al., 2020) reported that spike protein D614G SARS-CoV-2 variant demonstrates more efficient infection, replication, and competitive fitness than the wild type indicating that the evolution of the virus could make the drug and vaccine discovery efforts more challenging.

Here, we discussed the potency, safety, and pharmacokinetic profiles of drugs halting SARS-CoV-2 infection through targeting the M^pro^. Several drugs including alpha-ketoamide inhibitors, aldehyde-based inhibitors, N3, ebselen, carmofur, Feline coronavirus inhibitors (GC-373 and GC-376), GC-376 analogs, calpain inhibitors II and XII, and clinically approved anti-HCV and HIV drugs have been investigated for their potential anti-SARS-CoV-2 activity. Here we observed that most studies report only the *in vitro* potency and safety results while data on the *in vivo* pharmacokinetic profiles of potential drugs are very limited. Drugs **13a**, **13b**, ebselen, **11a** and **11b**, GC-376, GC-373, **6e**, boceprevir, narlaprevir, baicalein, remdesivir, calpain inhibitors II and XII, and UAWJ247 showed a very low EC_50_ value and a high CC_50_ value above 100 µM (except **6e** with a CC_50_ value below 65 µM) indicating their potency and safety. However, data on the *in vivo* pharmacokinetic profiles of new drugs were reported only for **13a**, **13b**, **11a,** and **11b**. Accordingly, although the currently available data are limited to decide the best new drug for further investigation, **11a** demonstrated better potency, safety, and *in vivo* pharmacokinetic activity (Table 1). Lack of sufficient data especially on new drugs hampered us to discuss the potency, safety, and pharmacokinetic characteristics of potential drugs impeding SARS-CoV-2 infection through inhibiting the M^pro^ in detail. Drug repurposing and the use of previously known drugs are very important to speed up the discovery of putative therapeutic options for new diseases during urgent times. As data on the pharmacokinetic profiles of known drugs are comparatively available, trying this option could be ultimately helpful provided that their suitability for COVID-19 patients should be determined. Despite limited data on the pharmacokinetic profiles of drugs, this review provides a glimpse into choosing the best new and/or repurposed drugs for further investigation.

Generally, current therapeutic options proposed to treat COVID-19 are mostly based on the results of *in vitro* studies, observational studies, and clinical trials (Fernandes et al., 2020); perhaps, computational predictions also account for a big proportion of these studies. Specifically, most studies on drugs targeting the main protease of SARS-CoV-2 present only data related to the *in vitro* potency and safety while *in vivo* pharmacokinetic profiling is very limited. The main protease is a crucial enzyme for virus replication and maturation (Ziebuhr et al., 2000; Jain and Mujwar, 2020) and has a relatively conserved active site (Stoermer, 2020; Ullrich and Nitsche, 2020) which makes it considered as a potential drug target (Naqvi et al., 2020). Remarkably, there are promising baseline data on potential inhibitors of SARS-CoV-2 M^pro^. Therefore, future research on drugs targeting SARS-CoV-2 M^pro^ should escape from preliminary computational, *in vitro,* and *in vivo* studies and advance to preclinical and clinical applications. Besides, cautious use of known broad-spectrum drugs in terms of potency, safety, selectivity, suitability, and binding affinity is also recommended. More importantly, COVID-19 therapeutic studies should consider the emergence of new SARS-CoV-2 variants due to virus evolution as the occurrence of 1-2 mutations every month is estimated (Duchene et al., 2020).

## Author Contributions

HM conceived the topic and wrote the original draft. All authors read and approved the final draft.

## Funding

TJ is supported by the Strategic Priority Research Program of the Chinese Academy of Sciences (Grant No. XDB29030104), the National Natural Science Fund (Grant Nos.: 31870731 and 31971129), the Fundamental Research Funds for the Central Universities, and the 100 Talents Program of the Chinese Academy of Sciences. HM is supported by the University of Science and Technology of China scholarship program. DM is supported by ANSO scholarship. AM is supported with CSC scholarship.

## Conflict of Interest

The authors declare that the research was conducted in the absence of any commercial or financial relationships that could be construed as a potential conflict of interest.
