# Supplementary material for: Potency, Safety, and Pharmacokinetic Profiles of Potential Inhibitors Targeting SARS-CoV-2 Main Protease
Source: Front Pharmacol. 2021 Feb 1;11:630500. doi: 10.3389/fphar.2020.630500 (PMC7883113; doi:10.3389/fphar.2020.630500)
Supplement: Supplementary file 1 [file datasheet1.pdf]

Supplementary Table 1: Chemical structure of compounds with anti-SARS-CoV-2 M^{pro} activity

Name	Chemical formula	Molecule name	Chemical structure
N3	C35H48N6O8	benzyl (3S,6R,9S,E)-9-isobutyl-6-isopropyl-3-methyl-1-(5 methylisoxazol-3-yl)-1,4,7,10-tetraoxo-12-((2-oxopyrrolidin-3-yl)methyl)-2,5,8,11-tetraazapentadec-13-en-15-oate	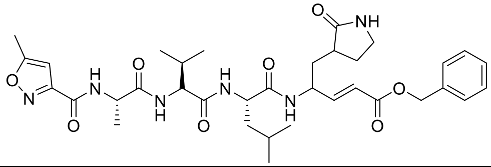
11a	C25H32N4O4	(~{N}-[(2~{S})-3-cyclohexyl-1-oxidanylidene-1-[[(2~{S})-1-oxidanylidene-3-[(3~{S})-2-oxidanylidenepyrrolidin-3-yl] propan-2-yl] amino]propan-2-yl]-1~{H}-indole-2-carboxamide	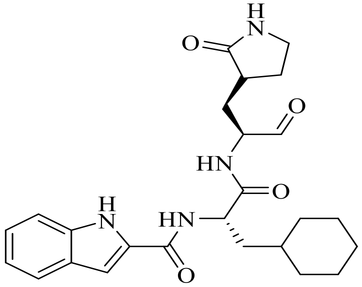
11b	C25H25FN4O4	~{N}-[(2~{S})-3-(3-fluorophenyl)-1-oxidanylidene-1-[[(2~{S})-1-oxidanylidene-3-[(3~{S})-2-oxidanylidenepyrrolidin-3-yl]propan-2-yl]amino]propan-2-yl]-1~{H}-indole-2-carboxamide	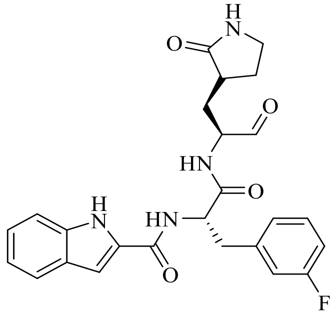
13b	C31H41N5O7	{tert}-butyl ~{N}-[1-[(2~{S})-3-cyclopropyl-1-oxidanylidene-1-[[(2~{S},3~{R})-3-oxidanyl-4-oxidanylidene-1-[(3~{S})-2-oxidanylidenepyrrolidin-3-yl]-4-[(phenylmethyl)amino]butan-2-yl]amino]propan-2-yl]-2-oxidanylidene-pyridin-3-yl]carbamate	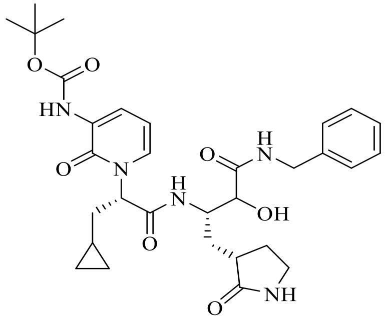

Baicalein	C15H10O5	5,6,7-trihydroxy-2-phenyl-4H-chromen-4-one	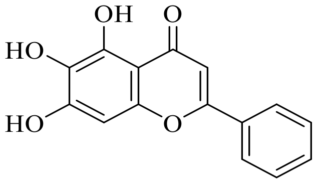
Boceprevir	C27H47N5O5	(1R,2S,5S)-N-[(2S,3R)-4-amino-1-cyclobutyl-3-hydroxy-4-oxobutan-2-yl]-3-[N-(tert-butylcarbamoyl)-3-methyl-L-valyl]-6,6-dimethyl-3-azabicyclo[3.1.0]hexane-2-carboxamide	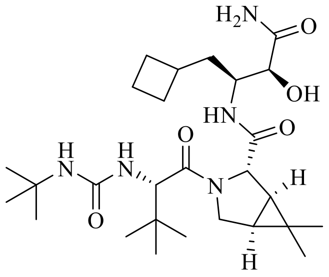
Narlaprevir	C36H63N5O7S	(1R,2S,5S)-3-[N-({1-[(tert-butylsulfonyl)methyl]cyclohexyl}carbamoyl)-3-methyl-L-valyl]-N-{(1S)-1-[(1R)-2-(2yclopropyl amino)-1-hydroxy-2-oxoethyl]pentyl}-6,6-dimethyl-3-azabicyclo[3.1.0]hexane-2-carboxamide	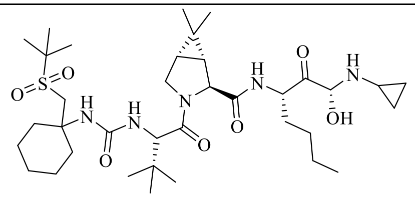
Telaprevir	C36H55N7O6	(1S,3Ar,6As)-2-[(2S)-2-({(2S)-2-cyclohexyl-2-[(pyrazin-2-ylcarbonyl)amino]acetyl}amino)-3,3-dimethylbutanoyl]-N-[(2R,3S)-1-(cyclopropylamino)-2-hydroxy-1-oxohexan-3-yl]octahydrocyclopenta[c]pyrrole-1-carboxamide	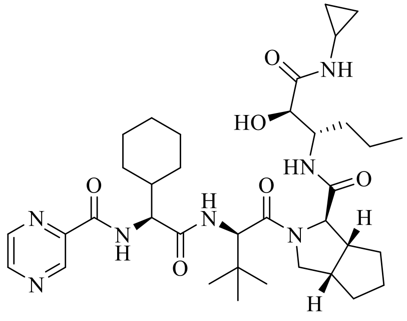

Simeprevir	$C_{38}H_{47}N_5O_7S_2$	(1*R*,4*R*,6*S*,7*Z*,15*R*,17*R*)-*N*-cyclopropylsulfonyl-17-[7-methoxy-8-methyl-2-(4-propan-2-yl-1,3-thiazol-2-yl)quinolin-4-yl]oxy-13-methyl-2,14-dioxo-3,13-diazatricyclo[13.3.0.0^{4,6}]octadec-7-ene-4-carboxamide	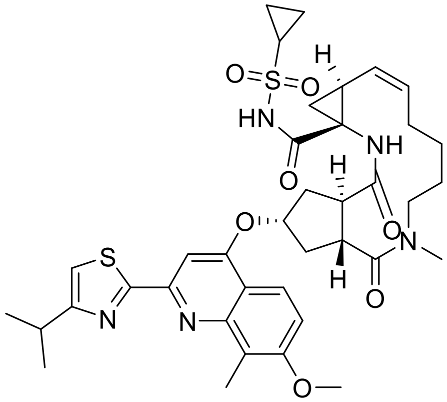
Grazoprevir	$C_{38}H_{50}N_6O_9S$	(1*R*,18*R*,20*R*,24*S*,27*S*)-24-*tert*-butyl-*N*-[(1*R*,2*S*)-1-(cyclopropylsulfonylcarbamoyl)-2-ethenylcyclopropyl]-7-methoxy-22,25-dioxo-2,21-dioxa-4,11,23,26-tetrazapentacyclo[24.2.1.0^{3,12}.0^{5,10}.0^{18,20}]nonacosa-3,5(10),6,8,11-pentaene-27-carboxamide	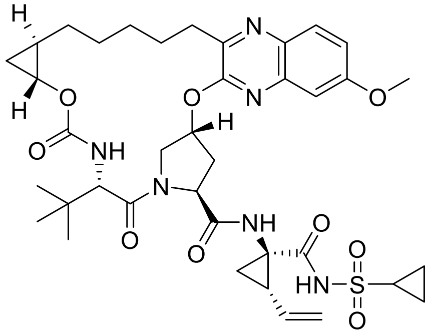
Carmofur	C7H15NO2	hexylcarbamic acid	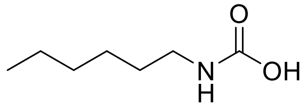
GC373	C21H31N3O5	N~2~-[(benzyloxy)carbonyl]-N-{(2S)-1-hydroxy-3-[(3S)-2-oxopyrrolidin-3-yl]propan-2-yl}-L-leucinamide	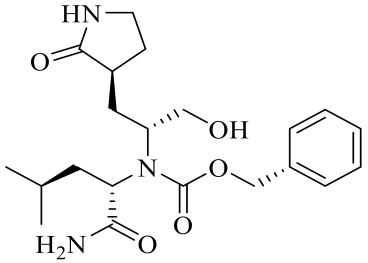

GC376	C21H31N3O8S	(1S,2S)-2-({N-[(benzyloxy)carbonyl]-L-leucyl}amino)-1-hydroxy-3-[(3S)-2-oxopyrrolidin-3-yl]propane-1-sulfonic acid	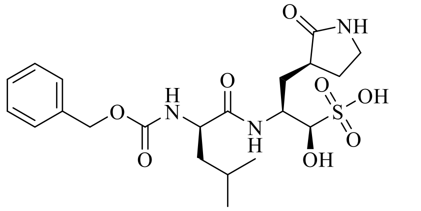
Lopinavir	$C_{37}H_{48}N_4O_5$	(2*S*)-*N*-[(2*S*,4*S*,5*S*)-5-[[2-(2,6-dimethylphenoxy)acetyl]amino]-4-hydroxy-1,6-diphenylhexan-2-yl]-3-methyl-2-(2-oxo-1,3-diazinan-1-yl)butanamide	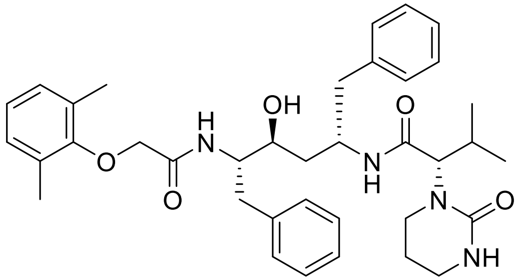
Nafamostat	$C_{19}H_{17}N_5O_2$	(6-carbamimidoylnaphthalen-2-yl) 4-(diaminomethylideneamino)benzoate	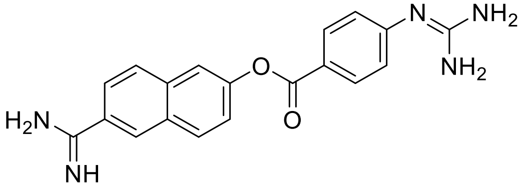
Remdesivir	$C_{27}H_{35}N_6O_8P$	2-ethylbutyl (2*S*)-2-[[[(2*R*,3*S*,4*R*,5*R*)-5-(4-aminopyrrolo[2,1-f][1,2,4]triazin-7-yl)-5-cyano-3,4-dihydroxyoxolan-2-yl]methoxy-phenoxyphosphoryl]amino]propanoate	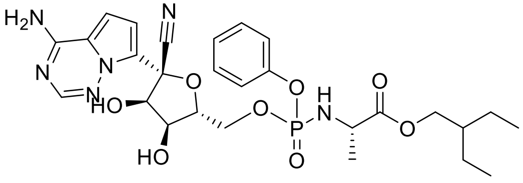
Ritonavir	$C_{37}H_{48}N_6O_5S_2$	1,3-thiazol-5-ylmethyl *N*-[(2*S*,3*S*,5*S*)-3-hydroxy-5-[[(2*S*)-3-methyl-2-[[methyl-[(2-propan-2-yl-1,3-thiazol-4 yl)methyl]carbamoyl]amino]butanoyl]amino]-1,6-diphenylhexan-2-yl]carbamate	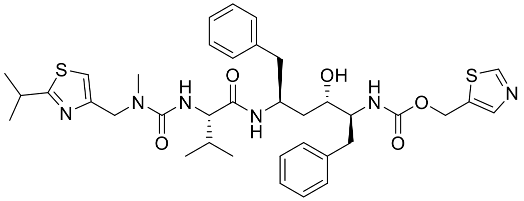

Imatinib	$C_{29}H_{31}N_7O$	4-[(4-methylpiperazin-1-yl)methyl]-*N*-[4-methyl-3-[(4-pyridin-3-ylpyrimidin-2-yl)amino]phenyl]benzamide	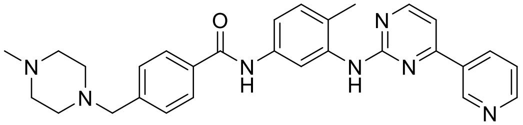
Ribavirin	$C_8H_{12}N_4O_5$	1-[(2R,3R,4S,5R)-3,4-dihydroxy-5-(hydroxymethyl)oxolan-2-yl]-1,2,4-triazole-3-carboxamide	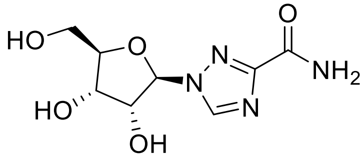
Rupintrivir	$C_{31}H_{39}FN_4O_7$	ethyl (*E*,4*S*)-4-[[(2*R*,5*S*)-2-[(4-fluorophenyl)methyl]-6-methyl-5-[(5-methyl-1,2-oxazole-3-carbonyl)amino]-4-oxoheptanoyl]amino]-5-[(3*S*)-2-oxopyrrolidin-3-yl]pent-2-enoate	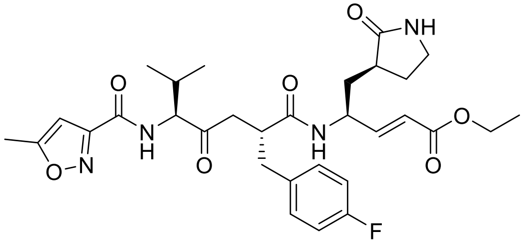
Ebselen	$C_{13}H_9NOSe$	2-phenyl-1,2-benzoselenazol-3-one	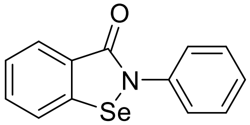
Cinanserin	$C_{20}H_{24}N_2OS$	(*E*)-*N*-[2-[3-(dimethylamino)propylsulfanyl]phenyl]-3-phenylprop-2-enamide	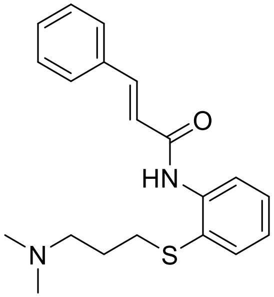
